# Open and impactful academic publishing

**DOI:** 10.3389/frma.2025.1544965

**Published:** 2025-03-26

**Authors:** Rosaria Ciriminna, Giovanna Li Petri, Giuseppe Angellotti, Rafael Luque, Mario Pagliaro

**Affiliations:** ^1^Istituto per lo Studio dei Materiali Nanostrutturati, National Research Council (CNR), Palermo, Italy; ^2^Universidad ECOTEC, Guayaquil, Guayas, Ecuador

**Keywords:** open access, self-archiving, open science, academic publishing, green OA

## Abstract

**Introduction:**

The advantages of self-archiving research articles on institutional repositories or personal academic websites are numerous and relevant for society and individual researchers. Yet, self-archiving has been adopted by a small minority of active scholars.

**Methods:**

Aiming to further inform educational work on open and impactful academic publishing in the digital era, we posed selected questions to Stevan Harnad 30 years after his "subversive proposal" to maximize research impact by self-archiving scholarly articles in university-hosted or disciplinary online repositories to make published articles openly available.

**Results and discussion:**

Self-archiving is even more needed today than it was when Professor Harnad called for it when the World Wide Web was in its infancy; OA academic publishing is a necessary but not sufficient condition for impactful research; self-archiving on a personal academic website is often more effective than in institutional repositories.

## 1 Introduction

In mid 1994 Harnad, a psychology scholar specializing in cognitive science at that time based at Princeton and Southampton universities, published on the VPIEJ-L Internet discussion list based at Virginia Polytechnic Institute a proposal for a conference presentation. Entitled “Publicly retrievable FTP archives for esoteric science and scholarship: a subversive proposal” (Harnad, 1994), the text called on all authors of research papers to self-archive their articles (the authors' refereed final drafts) for free in online archives accessible via file transfer protocol (FTP, a standard communication protocol used for the transfer of computer files from a server to a client on a computer network). Its *incipit* swiftly renders the practical approach praised by Harnad:

“We have heard many predictions about the demise of paper publishing, but life is short and the inevitable day still seems a long way off. This is a subversive proposal that could radically hasten that day (Harnad, 1994)”.

The post will originate the so-called open access (OA) movement in scholarly publishing (Craig et al., 2022). Research articles indeed were made available by publishers to readers via subscription or by paying an access fee. “The authors of refereed journal articles write them only to reach researchers and to have an effect on their research,” Harnad subsequently wrote, “the idea that access to them should be toll-gated in any way makes as much sense as tollgated access to commercial advertisements” (Harnad, 2002). In the same year, the scholar was invited to participate in a 2-day meeting convened in Budapest by the Open Society Institute (today Open Society Foundations) with the purpose “to accelerate progress in the international effort to make research articles in all academic fields freely available on the internet.” The meeting ended with the 16 participants signing a declaration (the Budapest Open Access Initiative, BOAI) soliciting researchers to make their research papers OA and free to reuse provided that authors retained “control over the integrity of their work and the right to be properly acknowledged and cited” (Open Society Institute, 2002). In the subsequent years, self-archiving research articles on a personal academic website, in a disciplinary repository like arXiv or on the website of the institutions employing the authors of research papers will be called by Harnad “green” OA (Harnad et al., 2008). The cognitive scientist will instead term “gold” OA, the other main form of open access in which authors pay an article processing charge (APC) to the publisher in exchange for managing the peer review and eventually openly publish online the refereed study (Harnad, 2010).

Given the subsequent proliferation of non-descriptive color names for different kinds of open access publishing (“blue,” “bronze,” “black,” “yellow” etc.), in 2022 Craig and co-workers made a strong argument against using unhelpful color names for different kinds of OA publishing, recommending to use descriptive names to describe different kinds of publishing (Craig et al., 2022). In the following, we adhere to this recommendation avoiding the use of color names for different types of OA, with the exception of “gold” OA.

Following conversation with Professor Harnad 30 years after the “subversive proposal,” this work answers three research questions:

RQ1: Is self-archiving still a valid option to maximize research impact?
RQ2: Besides providing open access, what is required for publishing impactful research articles?
RQ3: Is it better self-archive on a personal academic website or institutional repository?

## 2 Results and discussion

### 2.1 A conversation with Stevan Harnad

**First question is personal. You were amid the attendants of a meeting in Budapest in 2001 that is considered the debut of the open access/open science movement. What brought you there?**

I had already been advocating “OA” (before the name) for well over a decade, including my 1994 “Subversive Proposal” that all researchers should self-archive their refereed research online, free for all, and the American Scientist Open Access Forum (as of 1998). (This eventually was eventually dubbed “Green OA”).

I had also founded and edited one of the first “Gold” OA journals in 1989, *Psycoloquy*, sponsored by the American Psychological Association, and free for all. (“Gold OA”—which means the publisher makes the article OA, but it does not necessarily mean author-pays fees). And I had already been editor of an *Open Peer Commentary* journal, *Behavioral and Brain Sciences*, published by Cambridge University Press, since I founded it in 1978. (*Open Peer Commentary* was my real motivation for OA). I was invited to BOAI by Peter Suber, who had also been advocating OA (before the name) for some time. The name “OA” first appeared in Peter Suber's BOAI Declaration.

**What drove your attention to open access?**

*Open Peer Commentary*.

**Was it a personal dissatisfaction as a psychology scholar with the fact that most research articles were published and thus available only behind a paywall? Or was it a feeling of lack of social justice in light of the huge profits of scholarly publishers who had conquered who actually took for nothing new knowledge from unaware scholars seeking publication, and thus impact for their science?**

Scholars and scientists publish their peer-reviewed research articles to make them accessible to all potential users, to be read, used, applied, cited and built-upon. Their careers (“publish or perish”) also depend on this. It was hence obvious then, and still is now, that these give-away researchers, who seek and receive no revenue from their published journal articles, should make them accessible to all would-be users for free online.

**In 1994 you authored and published online the “Subversive Proposal” calling on scholars to archive their articles for free for everyone online. More than 30 years later, most scholars continue not to self-archive online their articles regardless of publishers granting permission to self-archive?**

I don't know about “most” anymore, in the online era, but I would say “not nearly enough.” (Publisher “permission” was never needed; belief in that was superstition, timidity, and rationalization all along).

**Why, in your opinion, has this happened?**

You mean why has it not happened nearly enough, nor fast enough.

**Is the delay due to lack of attention and digital skills of scholars being unable to open a personal academic website and post online their own articles?**

Initially, perhaps, though we at Southampton had already created EPrints (soon emulated by DSpace) free software so universities could create their own Green OA repositories. But today just about everyone has the digital skills and resources.

**Or has it been due to universities and other research institutions who were lazy and slow in making their websites also repositories of the articles published by their affiliates?**

Yes, that too. And most important, too lazy, slow, pedantic and timid to mandate deposit immediately upon acceptance for publication of the peer-reviewed final version of all research publications.

**The aim of making a scientific study open access, you wrote, is to maximize impact and uptake. Linacre has just published data showing that after “Green and Gold running neck-and-neck until they diverged over the last decade or so” showing that Green OA would have “failed in comparison to Gold Open Access”** (Linacre, 2023). **Yet, a recent analysis of Maddi and Sapinho comparing the citation impact of 2,458,378 publications in fully OA journals to that of a control group of non-OA publications over the period 2010–2020** (Maddi and Sapinho, 2022)**, found that there is no open access citation advantage for publications in fully OA journals and that there is rather a disadvantage. Hence, you were right: green self-archiving provides more benefits and increases impact, whereas “gold” OA does not. How do you explain similar findings?**

This is all non-sense. If an article is good, and useful, and researchers want it, they will access it any way they can (subscription, green or gold), read it, use it, apply it, and cite it. Open Access (whether Green or Gold) can only add to Toll Access usage, not reduce it. Access is access. The rest is about whether the research is worth reading, using, applying, and citing.

**A wise and purposeful use of “social media” by scholars seems to greatly benefit the societal impact of a scholar's research. How should a scholar practicing open science approach social media in your regarded opinion?**

Do good research, make sure to have it peer-reviewed and published, make it Green OA, and, if relevant, cite and discuss it in online peer discussion groups. But serious scholars and scientists are dedicated to the usage and uptake of their findings, not to their promotion on blogs, twitter, Reddit, or Ted Talks. We do not seek “Likes” but peer uptake, applying. And building upon it, to contribute to scholarly and scientific knowledge and learning (some of which stands among the few things the human species need not be ashamed of).

**One important journal in the life sciences, eLife, no longer accepts or rejects papers after peer review: all submissions that are peer reviewed are published as Reviewed Preprints after the peer-review process has been completed** (Eisen et al., 2020). **Do you still ascribe value to peer-review (and editing) as quality control means, as stated in the “Subversive Proposal”?**

This is just labeling and marketing. A paper that has been peer-reviewed to meet the standards of a reputable journal is published and can be cited as published in that journal. If it is not peer-reviewed, but “appears in” that journal, it is not a published (refereed-) journal article. It is merely an unrefereed journal, hosted by the journal's website.

**Why create confusion in users by making it harder to know what is and is not a peer-reviewed journal article?**

Yes, I still think it is important for researchers to know whether a paper is likely to be reliable and worth their time to try to use, apply and build upon. (Copy-editing has become much less reliable these days and maybe it's not worth the effort or cost).

“Peer review” can of course vary in quality and reliability from journal to journal. That's why the journal's name and track-record is still an important marker. Many journals have standards so low that their articles amount to unrefereed preprints. (This is true of subscription journals as well as pay-to-publish Gold OA journals, but the latter contain many more predatory junk journals).

I do also wonder what percentage of published research is important enough to warrant the time and effort of peers to referee it. The reserve of available, qualified peer expertise is greatly overstretched today, and referees are selected by secretaries (“editorial assistants”) through literature searches and software that are making a farce of the refereeing process.

Only time (not I) can tell what effect this is having on research quality, and whether it is worthwhile anymore. Can the default be “unrefereed,” with the hope that the important and reliable subset will be signaled by open peer feedback and usage (as the open “postpublication” “peer” review advocates propose)?

**Along with Green OA came the preprint, explicitly mentioned in your “Subversive Proposal.” When research is complete and written, it is made freely available to the scholarly community and to the public via self-publication on the internet (today on a preprint platform, on an academic social network, yesterday—as you did with the Subversive Proposal—via “anonymous ftp”). Even in this case, some research communities have been highly receptive, with massive uptake of preprint publishing. Others, very reluctant, even in the very same “basic” sciences (i.e., compare Physics with Chemistry) though its benefits are today well-documented even for the academic career of scholars preprinting their research. Why, in your opinion, such strong differences?**

It's partly discipline or speciality differences in habits and needs, but partly also differences in content and substance. I, for example, had been worried about the potential danger of blurring the boundary between refereed and unrefereed reporting of findings in medicine, but I do not know the public health data on that.

Peer-review is a form of “regulation” of quality and reliability, as in the domain of food and medicine. It varies greatly in quality in any case, and is easily thrown to the four winds. But I don't want to be the one to suggest throwing regulation to the winds. Let others bear the historical responsibility for that. (Unchecked climate change has already shown what we reap from sowing a free-for-all of profit-driven “libertarianism”).

**Along with research outcomes indirectly measured by the number of research papers and their citations, proponents of open science wisely suggest to include in the evaluation of scholarship teaching and societal service. Do you agree with this broader scope approach to the evaluation of scholars in academic promotion and tenure processes? Do you have practices of such an approach in evaluating colleagues in Canada, Great Britain or in other countries?**

To my knowledge, the evaluation of scholarly and scientific “output” is pretty mechanical and superficial in all these countries: not maximizing quality but regressing on the mean. I suppose that is part of the inevitable wages of scale. But I think we could do better with the many actual and potential online metrics available—as a supplement to, but not a substitute for—qualified human evaluation, taking into account publications, usage, scholarship, teaching, applications, and even activism.

### 2.2 Is self-archiving still a valid option to maximize research impact?

**Figure 1:**
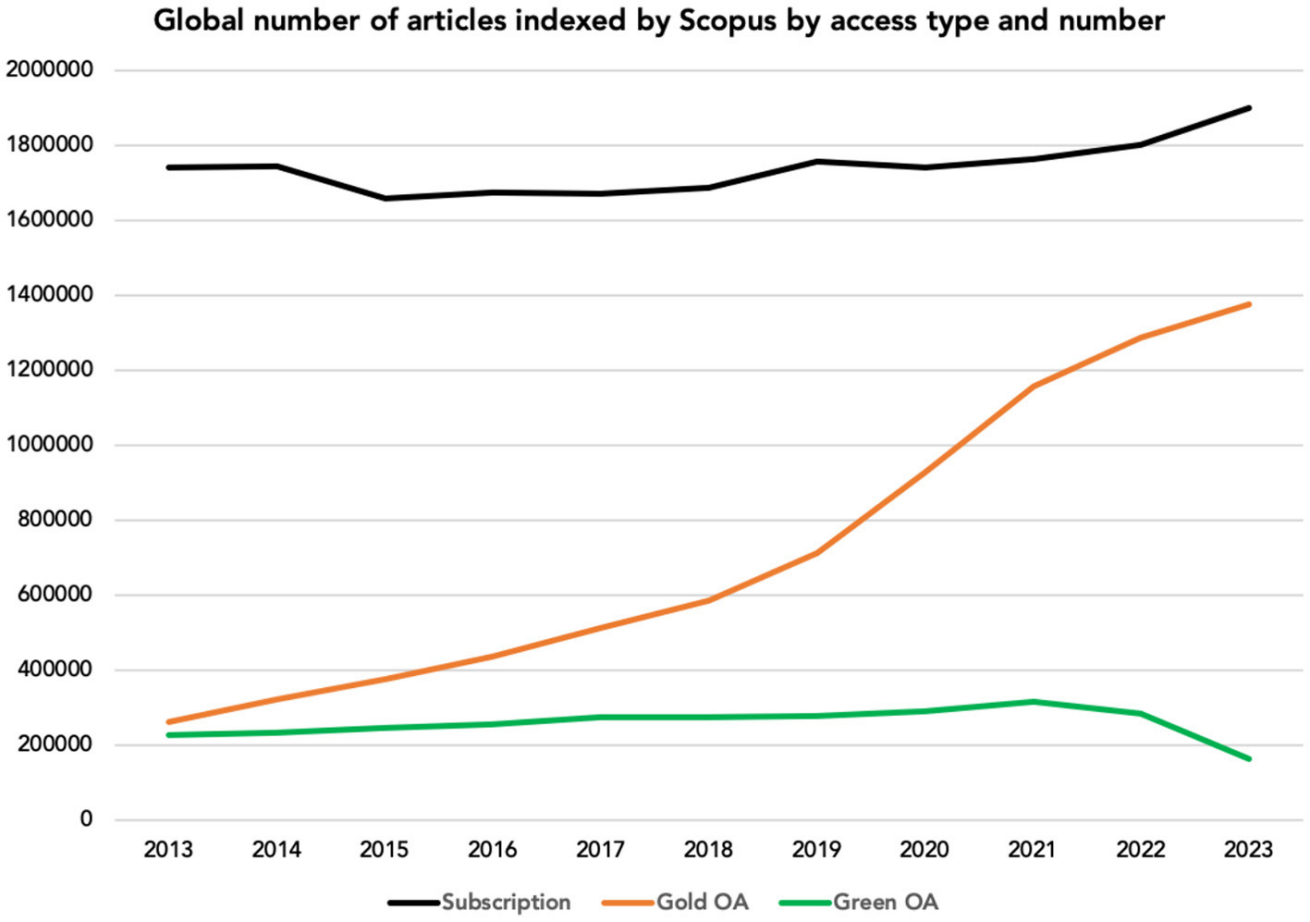
Global number of green OA, gold OA and paywalled articles indexed by Scopus by access type and number (Data source: Scopus, 2024 from STM, 2024).

In brief, charts in Figure 1 show how in 2023 the percentage of journal articles reviews and conference papers indexed by Scopus research database available to read via subscription-only amounted to 52%, further *increasing* with respect to 49% in 2019 and 51% in 2021 (STM, 2024). Furthermore, gold OA was by far the dominant type of open access, comprising 38% of all scholarly publications. Self-archived publications declined to 5% in 2023 from 9% in 2020 (STM, 2024). For comparison, in 2023 the number of publications self-archived amounted to 163,967, *vs*. 1,287,358 gold OA publications and 1,900.939 tollgated publications.

Relying on data from Dimensions, the most comprehensive research database (Singh et al., 2021), until 2013 the number of self-archived and gold OA articles indexed by Dimensions, was about 400,000 for both categories (Linacre, 2023). In the subsequent decade, however, numbers diverged with about 800,000 self-archived and 2,000,000 gold OA articles indexed in 2022. This outcome was due to government, university and funding agency policies action to support OA via direct publication in fully OA journals requiring payment of a fee as article processing charge (e.g., Plan S, “transformative agreements” between publishers and research institutions etc.). In brief, large-scale uptake of gold OA in wealthier countries (Japan, China, USA, western European countries and Russia) led to the decline of self-archiving in favor of gold OA. Linacre lately concluded that green OA “has failed” (Linacre, 2023).

Exploring the case for self-archiving in 2024 from a historic perspective, the answers of Harnad are revealing. The slow uptake of self-archiving was due both to: *i*) the researcher's belief (“superstition”) in a publisher permission to self-archive “that was never needed,” and *ii*) to universities and research institutes being “too lazy, slow, pedantic and timid to mandate deposit immediately upon acceptance for publication of the peer-reviewed final version of all research publications.” The psychologist also adds “rationalization” and “timidity” to the author behavioral traits that contributed to the aforementioned belief.

Skepticism about the benefits of OA for research impact and limited availability of university-hosted repositories, we further argue, are two other main drivers that may explain such counterintuitive author and university behavior. “Universities and their researchers” indeed in principle “share in the benefits of maximizing research impact and share in the costs of lost impact” (Harnad et al., 2008). Ever since the “subversive proposal” Harnad insisted that the main objective of OA was to maximize research impact, with the latter translating into further research funding, research progress and joint advances to the researcher's career and to financial support of the researcher's institution.

Put simply, skepticism was due to the fact that most researchers were (and still are) not familiar with the open access citation advantage (OACA) data published by Harnad and co-workers in 2008 (Harnad et al., 2008) using a 12-year sample of 14 million articles indexed by the Institute for Scientific Information database between 1992 and 2003 for the fields of physics, sociology, psychology, law, management, education, business, health sciences, political sciences, economics and biology. The team identified an OACA varying between 40% in biology and nearly 250% in physics.

Indeed, in the last 3 months of 2004, Swan and Brown carried out a cross-disciplinary study on green OA surveying 1,296 researchers (74% of whom were academics, 3% in research institutions, 5% in the public sector and 5% in industry or business) on self-archiving articles using personal web pages, institutional repositories and disciplinary repositories (Swan and Brown, 2005). More than half (51%) had never self-archived at least one article during the previous 3 years. Out of the 49% of the respondents who had self-archived articles and conference presentations, most researchers (27%) had opted for posting a copy of the refereed, published research article on a personal web page rather than on institutional (20%) or subject-based (12%) repositories. The share of researchers using their personal website had even increased (27%) when compared the 20% found in the previous survey (January 2004).

In 2008, the launch of academic social network ResearchGate (RG) gave place to the first substantial change. A significant number of scholars opted to use academic social networks such as RG (or Academia.edu) to self-archive their articles, without paying attention to copyright issues. “ResearchGate was able to garner early adoption” commented an entrepreneur and data scientist in 2023, “by providing a venue for authors to post their articles despite having relinquished the copyright to do so” (Himmelstein, cited in: Trager, 2023).

For example, in early 2015 RG already had 7 million members and 19 million full-text publications. Analysis of 500 papers randomly selected from a one million list of papers available as full-text found that only 108 out of 500 were OA articles, whereas 201 (51.3%) out of 392 non-OA articles uploaded infringed the publishers' copyright (Jamali, 2017). Publishers predictably reacted, and in 2017 two large publishers sued RG for providing unrestricted public access to copyrighted articles. Today, after having settled litigation with the publishers in late 2023, RG generally does not include non-OA articles uploaded by authors.

As expected, in order to prevent legal issues due to copyright infringement, universities and research institutes owning “institutional repositories” (usually the website of the university) opted to check permissions for any published faculty research paper prior to make it OA (Hanlon and Ramirez, 2021). As a result, “from the perspective of faculty members the time and effort involved in determining or securing copyright often outweighed the benefits of institutional repositories” (Jamali, 2017).

Things further accelerated since 2011, when the Sci-Hub online database was launched. In a few years, the database made *de facto* openly accessible nearly the whole scholarship output. As of March 2017, about 69% of the 81.6 million scholarly articles registered with Crossref and slightly more than 85% of articles published in paywalled journals were freely available on Sci-Hub (Himmelstein et al., 2018). Besides legal and ethical consideration (Sci-Hub provides free access to copyrighted articles), this simple fact, found Maddi and Sapinho in 2022, “instantly cancels the positive effect of OA publication insofar as question of access to scientific content no longer arises” (Maddi and Sapinho, 2022).

Indeed, following comparison in the citation impact of 1,024,430 publications in “hybrid” journals (subscription journals in which author can make their articles OA following payment of an article processing charge) to that of a control group of non-OA publications over the period 2010–2020, the two scholars found that the high (60%) OACA in 2010 reached its maximum 70% in 2016. Since then, it steadily declined to about 20% by 2020 (Maddi and Sapinho, 2022). Furthermore, following comparison in the citation impact of 2,458,378 publications in fully OA journals, the team identified an open access citation *disadvantage* for publications in fully OA journals amounting to about −20 to −15%, when compared to the uncontrolled group comprised of all non-OA publications indexed by the research database Web of Science in the same time framework (Maddi and Sapinho, 2022).

**Figure 2:**
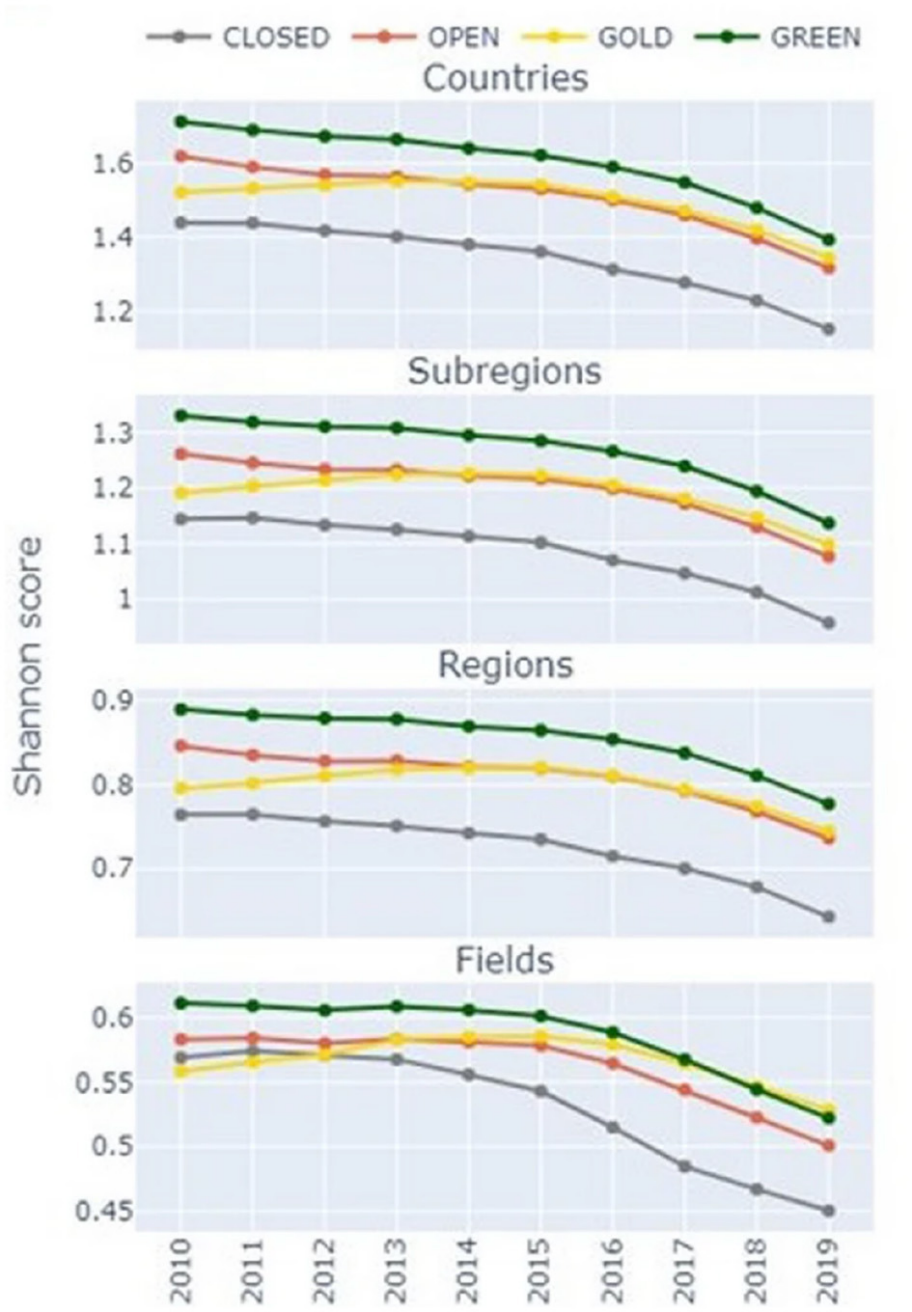
Mean Shannon scores compared across paywalled and OA category publications, with the scores calculated based on the grouping of citing affiliation links by countries, subregions, and regions, and citing outputs by fields of research over a 10-year period (2010–2019). (Reproduced from Huang et al., 2024, Creative Commons License 4.0).

A recent investigation of 19 million research outputs (and 420 million citation links) published from 2010 to 2019, found that green OA has a stronger effect on increased diversity of citation sources by institutions, countries, subregions, regions, and fields of research than gold OA via publisher platforms (Huang et al., 2024). Plots of the mean Shannon scores by citing sources compared across closed, open, gold OA and self-archived article categories between 2000 and 2019 showed that self-archiving benefits researchers because it allows to reach *wider audiences* (Figure 2). The Shannon diversity index, we briefly remind, is a measure of a population (citations, in this case) diversity derived from information theory.

In detail, comparison of paywalled and open publications by countries, subregions, and regions, and citing outputs by fields of research shows (Figure 2) that for the first three cases, all OA categories consistently outperform paywalled outputs, and that OA publications outperform paywalled outputs for the fields of research since 2013. Furthermore, green OA outperforms all open publication categories in *all* terms of diversity investigated across all years: country, subregion, and region of author affiliations and fields of research of citing outputs.

These outcomes, thus, reinforce the opportunity to self-archive research papers, rather than pursuing OA via publishing in gold OA journals.

### 2.3 Besides OA, what is required for publishing impactful research articles?

In general, even some scientific journals still exist in print and digital formats, most scholarly journals no longer print journals but only publish online the digital version of research papers in PDF format (to maintain the reader-friendly format developed for scholars who will print and read physical copies of research articles) or are transitioning to exclusively digital distribution. Articles often are published also in (“digital-first”) computer readable interactive markup languages such as HTML and full-text XML. This makes self-archiving on a personal academic website even more desirable.

Generally available as PDF files, most self-archived refereed research articles are available as single-column pages using double spacing, often with tables and figures placed at the end of the manuscript. This format, however, was the old manuscript format required by journals when peer review was carried out by reviewers by handwriting through the paper on manuscripts exchanged through postal service. Article templates allow authors to place images, graphs and tables close to the point where they are discussed within the text, can be easily accessed online and used to produce research papers directly in typeset format. This enhances readability (both on paper after printing the article and on screen), and increases the chances of engaging and retaining an audience amid peers. In 2012, for example, just over half (51.1%) of articles were found to be read on the screen of a computer or of a mobile digital device (Tenopir et al., 2015).

Format affects understanding at all levels. Even the in-text citation format affects reading comprehension, with undergraduate students (enrolled in science courses) reading comprehension being 34% higher when reading articles with numeric in-text citations compared to articles with parenthetical, Author-Year, in-text citations (Cowan et al., 2020). Research papers, furthermore, are read by peers by first reading the abstract and the conclusions (Subramanyam, 2013). Hence, authors of impactful papers will both use a template to format their paper *and* learn how to write succinct abstracts clearly stating the scope of the investigation and the main conclusions after summarizing the main results. Empirical evidence studying the understanding of third-year undergraduate students clearly shows that this results in substantially higher readability, understanding, and confidence (Freeling et al., 2021). On the other hand, using the intrinsic multimedia communication capability of the world wide web, today several journals use visual abstracts, consisting of an infographic style format coupled with a short word summary of the research detailing the research questions, findings and take home message of the research study (Millar and Lim, 2022).

Academic publishing is a market where information is exchanged for attention (Franck, 2002). In this market information is plentiful and growing at fast pace whereas attention is the scarce resource. Since 1952, indeed, the number of research papers published annually grows exponentially with an annual growth rate of 5.08% and a doubling time of 14 years (Bornmann et al., 2021). Hence, any effective means to enhance scholarly attention, such as using visual abstracts to disseminate research using social media (Bonnevie et al., 2023), will enhance the chances of having the research being read, used and cited.

### 2.4 Self-archive on a personal academic website or institutional repository?

A personal academic website is “a free option that allows authors to aggregate their works in their own web space” that does not “come at a cost to manuscript discoverability” (Goben and Akers, 2020). Two major reasons support the opportunity to use a personal academic website (Ciriminna and Pagliaro, 2024) properly developed according to the principles and guidelines of search engine optimization to self-archive scholarly work.

First, self-archiving a research paper on a personal academic website saves time and eliminates bureaucracy making self-archiving quick and effective. For example, in France in 2020, even though self-archiving in the HAL repository was mandated for evaluation of CNRS researchers, archiving by non-faculty still accounted for 52% of all deposits (Schöpfel et al., 2023). Researchers in most world's countries continue to not self-archive even in scientifically leading countries such as the United States of America (USA). For instance, University of North Carolina at Charlotte's institutional repository at the end of 2021 hosted 126 works, despite the 3,000 faculty during that period had published 4,817 works (Lake and Regenauer, 2024). In the subsequent academic year, the librarians thus created a mediated workflow in which they would identify and deposit scholarly papers for the repository on behalf of the researcher. In academic year 2022–2023 out of a campus with 3,000 faculty, 14 faculty members used the workflow to add 158 works to the repository. Combined with independent self-archiving, the overall number of works self-archived at UNC Charlotte reached 276 in 2022–2023, from 123 in the previous academic year. In brief, faculty and researchers remain non-responsive. “This lack of response could be for a variety of reasons, including people feeling burdened by too many emails” (Lake and Regenauer, 2024).

Another reason for which a scholar should use his/her personal academic website is that for over two decades since the Web introduction, until collaboration between publishers and search engine companies was established, academic articles stored in publishers' databases were actually part of the “academic invisible web” (Lewandowski and May, 2006). Remarkably indeed, research conducted in 2006 on self-archiving amid assistant, associate, and full professors (a 1,500-member survey sample group of 17 doctorate-granting universities in the USA found that self-archiving on personal web pages was adopted for self-archiving by 67% of the faculty who self-archived works, with most surveyed faculty responding that “self-archiving required minimal time and effort” (Kim, 2010).

## 3 Conclusions

In summary, following Harnad's 1994 “subversive proposal” to self-archive published articles on the Internet (Harnad, 1994), the cognitive scientist and his co-workers demonstrated a large and significant open access citation advantage for all disciplines ranging from ~40% in biology to nearly 250% for articles in physics (Harnad et al., 2008). Addressing a letter to *Nature* in 2001, Lawrence did the same for papers in computer science: analysis of articles published in the same journal gave an increase of 286% in the citation rate for online articles (Lawrence, 2001). One would therefore expect that scholars worldwide would have embraced self-archiving *en masse*. This, however, did not happen, and in 2022 the ~800,000 self-archived articles indexed by the most comprehensive research database were less than half of the ~2,000,000 Gold OA articles (Linacre, 2023). This outcome also damages society, which directly supports academic research work through taxpayer money, in terms of delayed innovation (Probst et al., 2021).

To understand the cause of this behavior we posed selected questions to Stevan Harnad 30 years after his “subversive proposal.” The main drivers of this outcome were a combination of factors including unfounded skepticism concerning OA, and bureaucratic access to the few institutional repositories available. In brief, concomitant with the global trend that made citations of scholarly papers and other citation-based metrics the dominant evaluation criteria to evaluate researchers for hiring, tenure and promotion (Kulczycki, 2023), most scholars and universities across the world opted to deal with self-archiving as something chiefly concerning the advocates of “open science.” Similar skepticism was accompanied by unjustified prejudice toward the first fully OA journals (Krawczyk and Kulczycki, 2021).

As time passed, scholars became aware of the OA citation advantage. Yet, rather than making their articles freely accessible on institutional repositories or on personal websites, since 2009 they started to publicly upload their journal articles on academic social networks such as ResearchGate (Jamali, 2017). This lasted till late 2017 when, following legal action from publishers for copyright infringement, most articles posted on academic social websites were eventually removed (Himmelstein, cited in: Trager, 2023). In the meanwhile, the scholarly community based in economically developed countries started to uptake Gold OA, namely publication of articles in OA after payment to the publisher of a fee. Paradoxically, however, the success of the Sci-Hub platform illegally providing access to nearly the whole scientific literature has canceled the positive citation advantage of OA publishing, creating the conditions for a OA citation *disadvantage* for publications in fully OA journals (Maddi and Sapinho, 2022).

In this context, this study aimed at fostering further progress toward open and impactful academic publishing answers three relevant questions on self-archiving 30 years Harnad's “subversive proposal.”

The first answer is that, far from having “failed” (Linacre, 2023), self-archiving is even more needed today than it was when Harnad called for self-archiving, with the world wide web in its infancy. Self-archiving indeed shows a stronger effect on increased *diversity* of citation sources by institutions, countries, subregions, regions, and fields of research than gold OA, as shown by recent investigation of 19 million research outputs and 420 million citations published from 2010 to 2019 (Huang et al., 2024).

The second answer is that OA academic publishing is a necessary but not sufficient condition for impactful research. Self-archived research papers need to be written in a clear and concise style; *and* directly in reader-friendly format. This in its turn requires that universities provide undergraduate and graduate students with formal education on scientific writing, and on the principles and tools of open science.

The third answer is that self-archiving on a personal academic website is even more desirable today than it was (and is) self-archiving in institutional repositories because it saves time and eliminates bureaucratic burdens often associated with “posting” works on online repositories.

One decade after those identified a decade ago by Björk and co-workers (Björk et al., 2014), the findings of this study on research article self-archiving may inform the aforementioned (and highly needed) educational programs of universities on open and impactful academic publishing in the digital era (Pagliaro, 2020).

## Data Availability

The original contributions presented in the study are included in the article/supplementary material, further inquiries can be directed to the corresponding authors.
